# British Nursing Advancing: What "British Women's Hospital" Stands for

**Published:** 1917-10-27

**Authors:** 


### THE MATRONS' AND SISTERS' DEPARTMENT.

### BRITISH NURSING ADVANCING.

### What "British Women's Hospital" Stands For.

October in Great Britain has long meant for the Money Market, business enterprises generally, and the best administered of our greatest institutions the period of the year when new enterprises and important developments in progress and finance are launched. The action of our women since the advent of the Great War has brought them into line with men workers, and everywhere their claim to higher recognition and greater independence has been gratefully recognised and admitted. It is all in keeping with the times that the British Women's Hospital Organisation, of which Her Majesty the Queen and Her Majesty Queen Alexandra are the patrons, should at this period voice the wish and will of the nation in its desire to give expression to its pride, gratitude, and recognition of the debt owed to trained nurses and all who have ministered everywhere to the alleviation of the sufferings and the recovery of our fighting men and the civil population and their dependants.

Everyone who is interested in the war—and who of us is not?—must therefore welcome the British Women's Hospital appeal in aid of the Nation's Fund for Nurses, established as a Thank-Offering from the British Empire to British Nurses. The British Women's appeal on its merits is an able manifesto, and will be welcome to the people of the Empire of all classes. In commending it to the nation we have summarised its chief and most telling points. The Nightingale Fund in 1856, after the Crimean War, produced some £50,000. Now, the British troops in the Crimea, in whose sufferings the foundation of nursing was laid, never numbered more than 70,000 men. Hence the British Women now organising the Nation's Fund for Nurses, it will be agreed, are justified in believing that the Tribute Fund for Nurses issued by British Women on October 22, 1917, when the British Armies at Home and in every zone of the far-flung battle-line are counted in millions, and British trained nurses number tens of thousands of women, can readily and speedily yield a quarter of a million pounds. It is properly claimed that "the greatness of this Memorial lies in the opportunity for development which it offers to the nursing profession. Almost everyone at some time has had reason for gratitude to the devoted women on whom it rests." Doubtless there have been moments in the lives of all when they have felt an instinctive desire to prove their gratitude to the trained nurse. The British Women's Tribute from the British Nation to British Nursing gives all their opportunity.

The Memorial will assume a double form. **First,** it will materially aid the reorganisation of the nursing profession by the nurses themselves, with the aid of those who, by experience and up-to-date knowledge, have the interests of the nurses at heart. A College of Nursing is therefore in being, is making its influence felt, and branches have already been formed in Scotland and Ireland. But the scope of the College is not bounded by the confines of the British Isles; its outlook is Imperial; it aims at extending its privileges and usefulness by means of affiliated branches to the far-flung Dominions overseas, which have never been nearer to the Empire than they are at the present time, when our sons have fought and bled and died together. In British nursing there is at present no recognised standard of education. There is no protection to the public against the half-trained or worthless woman. The profession has no headquarters to which employer and employed alike can turn for counsel and help. The emergencies of war conditions, which affected nursing in all its branches, and which called for faultless machinery to meet the difficulties, found the nursing profession unorganised, unprepared, and with no central authority empowered to direct, control, and mobilise it so that it might be used to the best advantage for the country.

The British Women hope that out of this disorganisation—accentuated by the war—a National Memorial may be erected for the nursing profession. But no institution of this kind can live without money, and no organisation can hope to get money unless the object for which that money is required is made perfectly clear. The British Women, therefore, appeal for an Endowment Fund. Other professions have appealed successfully for funds for the endowment of their colleges, and the College of Nursing is designed, in the first instance, to stand in the same relation to the profession of nursing as the Colleges of Physicians and Surgeons have to the professions of Medicine and Surgery. In order that it may fulfil this design it requires of necessity an Endowment Fund. To fulfil its mission properly the College should be in a position to offer every inducement and facility to those who show special aptitude for the higher branches of nursing. The provision of scholarships enabling their holders to pursue special courses of study or to travel with a view to acquainting themselves with methods which govern nursing practice in other countries are steps in the same direction. All sorts of studentships and post-graduate courses will be required, and those donors who prefer to give sums for purposes of specific endowment or studentships can attach their name permanently to them. It is hoped, too, that money may be forthcoming to provide a suitable building in London for the headquarters of the College, with examination, lecture, and club rooms where nurses may meet.

**Secondly,** the British Women are ambitious to found a Tribute Fund in addition, so that the

College of Nursing may have ample funds, for it may have to consider many tragedies arising out of the war, owing to the great risks, mental and physical, which nurses run—loss of sight, injury to limbs, nervous breakdown from shock and overwork, and other serious maladies. The nursing profession tests the endurance of its members very severely, and not all have the strength to suffer its hardships without mishap. There are few years in a nurse's life when her earnings are sufficient to enable her to save adequately for illness or old age, and up to the present the profession on the whole has been miserably under-paid. The standardisation and organisation of the profession through the College of Nursing must provide a remedy for these evils, and the British Women hope that the generosity of the public may enable the College, through the Tribute Fund, to make provision for such of its members, through no fault of their own, who may be in need or in suffering.

The British Women's Hospital appeal offers an attractive opportunity for every section of the nation to render back to the nurses themselves a little of the immeasurable debt owed to them by the community. We have here given a terse summary of the main grounds on which the British Women rest their plea to win the support of the British nation, and to secure for these popular and most attractive objects a representative gift from the British People of £250,000.

